# Caesium-rich micro-particles: A window into the meltdown events at the Fukushima Daiichi Nuclear Power Plant

**DOI:** 10.1038/srep42731

**Published:** 2017-02-15

**Authors:** Genki Furuki, Junpei Imoto, Asumi Ochiai, Shinya Yamasaki, Kenji Nanba, Toshihiko Ohnuki, Bernd Grambow, Rodney C. Ewing, Satoshi Utsunomiya

**Affiliations:** 1 Department of Chemistry, Kyushu University, Motooka 744, Nishi-ku, Fukuoka 819-0395, Japan; 2 Faculty of Pure and Applied Sciences and Center for Research in Isotopes and Environmental Dynamics, University of Tsukuba, 1-1-1 Tennodai, Tsukuba, Ibaraki 305-8577 Japan; 3 Department of Environmental Management, Faculty of Symbiotic System Science, Fukushima University, Kanayagawa 1, Fukushima, 960-1296 Japan; 4 Laboratory for Advanced Nuclear Energy, Institute of Innovative Research, Tokyo Institute of Technology, 2-12-1 Ookayama, Meguro-ku, Tokyo 152-8550, Japan; 5 SUBATECH, IMT Atlantique, Université de Nantes, CNRS/IN2P3, Nantes 44307, France; 6 Department of Geological Sciences and Center for International Security and Cooperation, Stanford University, Stanford, CA 94305-2115 USA

## Abstract

The nuclear disaster at the Fukushima Daiichi Nuclear Power Plant (FDNPP) in March 2011 caused partial meltdowns of three reactors. During the meltdowns, a type of condensed particle, a caesium-rich micro-particle (CsMP), formed inside the reactors via unknown processes. Here we report the chemical and physical processes of CsMP formation inside the reactors during the meltdowns based on atomic-resolution electron microscopy of CsMPs discovered near the FDNPP. All of the CsMPs (with sizes of 2.0–3.4 μm) comprise SiO_2_ glass matrices and ~10-nm-sized Zn–Fe-oxide nanoparticles associated with a wide range of Cs concentrations (1.1–19 wt% Cs as Cs_2_O). Trace amounts of U are also associated with the Zn–Fe oxides. The nano-texture in the CsMPs records multiple reaction-process steps during meltdown in the severe FDNPP accident: Melted fuel (molten core)-concrete interactions (MCCIs), incorporating various airborne fission product nanoparticles, including CsOH and CsCl, proceeded via SiO_2_ condensation over aggregates of Zn-Fe oxide nanoparticles originating from the failure of the reactor pressure vessels. Still, CsMPs provide a mechanism by which volatile and low-volatility radionuclides such as U can reach the environment and should be considered in the migration model of Cs and radionuclides in the current environment surrounding the FDNPP.

The nuclear disaster at the Fukushima Daiichi Nuclear Power Plant (FDNPP) in March 2011 caused partial meltdowns of three reactors^1^,^2^,^3^, which caused the second-most serious nuclear accident in history^4^, resulting in serious environmental threats with the release of ~5.2 × 10^17^ becquerels (Bq) of radionuclides^5^ in Fukushima prefecture. Many of the resulting problems, including radioactive caesium contamination of the surface environment, have yet to be resolved^6^. The most challenging issue remaining is the treatment of the four damaged reactors. Decommissioning of Units #1–4 is currently ongoing^7^, although the properties of the melted fuels mixed with reactor components, referred to as debris, and the conditions inside the reactors remain unknown because the high-radiation field prevents access^7^.

Until now, the reactions that occurred in the FDNPP reactors have been only inferred based on indirect evidence^3^. It is believed that radioactive Cs was liberated from the irradiated fuel when the temperature of the fuel rose above 2,200 K^8^ after the cooling systems shut down in Units #1–3. Other radionuclides were released in amounts depending on their respective volatilities^9^ rather than in amounts based on their estimated presence in the nuclear fuel, which was primarily composed of UO_2_^8^,^10^. Thus, a large portion of the fission products (FPs), including radioactive Cs still remain in the damaged reactors and in contact with the cooling water^11^. To carry out an adequate decommissioning process, it is of critical importance to understand the physical and chemical state of the radionuclides inside the reactors^12^. In particular, most of the irradiated fuels melted in Units #1 and #3, while a lesser amount or none of the fuels underwent melting in Unit #2^3^,^13^. Melted fuel accumulated at the bottom of the reactor pressure vessels (RPVs), which eventually caused the RPVs to rupture, leading to reactions with the concrete pedestals of the primary-containment vessels (PCVs)^14^, a process known as molten core concrete interaction (MCCI)^15^. There remains considerable uncertainty about the extent of the MCCI in the reactors and the state of the melted fuel.

Caesium-rich micro-particles (CsMPs) originating from the FDNPP were first found in atmospheric particles some 170 km southwest of the FDNPP^16^,^17^. These particles represent condensed matter that formed within the reactors during meltdown, and they provide important information on the physical and chemical characteristics of the radioactive material inside the reactors. This study unravels the formation process of the CsMPs based on their chemical and structural properties at the atomic scale utilizing a high-resolution transmission electron microscopy (HRTEM) in conjunction with conventional radio-analytical techniques.

## Methods

### Sample description

The sampling campaign was conducted on 16 March 2012. The Ottozawa soil sample (OTZ) was collected from the top ~1 cm of soil in a paddy located ~4 km west of the FDNPP in Okuma Town, Futaba County, Fukushima. The soil was primarily composed of clay minerals, quartz and feldspars. Because entering the area was still restricted due to the high radiation dose, the locality had not been artificially disturbed. The radiation dose ~1 m above the ground was 84 μSv/h. The gravel sample from Koirino (KOI) was collected under the drainpipe of the assembly house. The house is located 2.9 km southwest of the FDNPP. The radiation dose beneath the drainpipe was extremely high compared with the surroundings, with a sampling area dose as high as 630 μSv/h. The gravel samples were carefully collected from the surface of the ground using a hand shovel, and placed in plastic bags. The aquaculture centre (AQC) soil samples were collected from the side ditch of an aquaculture centre located ~2 km south of the FDNPP.

### Separation of CsMPs

The procedure for separating CsMPs from the soil samples is schematically illustrated in Fig. S1. Prior to the procedure, both samples were sieved through a 114 μm mesh. The powder samples were dispersed on grid paper and covered with a plastic sheet, and an imaging plate (Fuji film, BAS-SR 2025) was placed on the samples for 5–25 min. Autoradiograph images with pixel sizes of 50–100 μm were recorded using an imaging-plate reader. After the positions of intensely radioactive spots were identified, droplets of pure water were added to these positions and then drawn using a pipette to produce suspensions with small amounts of soil particles by dilution with pure water (Procedures 3–9 in Fig. S1). This procedure was repeated until the suspension did not contain a significant amount of soil particles. Subsequently, positions containing hot spots were selected using pieces of double-stick carbon tape that were cut as small as possible with a blade. The pieces of tape were checked by autoradiograph imaging so that scanning electron microscopy (SEM) observation could be performed to obtain the CsMPs with maximum efficiency. Prior to SEM analysis, the pieces of tape were placed on an aluminium plate and coated with carbon using a carbon coater (SANYU SC-701C). The CsMPs were found using an SEM (Shimadzu, SS550 and Hitachi, SU6600) equipped with an energy dispersive X-ray spectrometer (EDX, EDAX Genesis) using acceleration voltages of 5–25 kV for imaging details of the surface morphology and 15–25 kV for elemental analysis, including area analysis and elemental mapping.

### Preparation of the TEM specimen

A focused ion beam (FIB) instrument (FEI, Quanta 3D FEG 200i Dual Beam) was utilised to prepare a thin foil of individual CsMPs with diameters of a few μm. Gallium was used as an ion source, and W deposition was used to minimise damage from the ion bombardment. Prior to application of the FIB, each SEM specimen was coated with ~40 nm-thick gold. The current and accelerating voltage of the ion beam were adjusted from 100 pA to 30 nA and 5–30 kV, respectively, depending on the progress of the thinning and on sample properties such as hardness and size. Each thinned piece was attached to the semilunar-shaped Cu grid for FIB and further thinned by an ion beam operating at 5 kV.

### TEM analysis

HRTEM with EDX and a high-angle annular dark-field scanning transmission electron microscopy (HAADF-STEM) were performed using a JEOL JEM-ARM200F and JEM-ARM200CF with an acceleration voltage of 200 kV. The JEOL Analysis Station software was used to control the STEM-EDX mapping. To minimise the effects of sample drift, a drift-correction mode was used during acquisition of the elemental map. The STEM probe size was ~0.13 nm, generating ~140 pA of current when 40 μm of the condenser lens aperture was inserted. The collection angle of the HAADF detector was ~97–256 mrad.

### Gamma spectrometry

The ^134^Cs and ^137^Cs radioactivities of the CsMPs were determined using gamma spectrometry. The radioactivity of an additional micro-particle with a size of ~400 μm obtained from the surface soil in Fukushima was precisely determined at the radioisotope centre in Tsukuba University, Japan, and utilised as a standard point specimen for ^134^Cs and ^137^Cs. The radioactivity of the point source standard was 23.9 Bq for ^134^Cs and 94.6 Bq for ^137^Cs as of 29 September 2015. The measurement of radioactivity was performed on the CsMPs and the point source standard using germanium semi-conductor detectors GMX23, GMX30 and GMX40 (all from SEIKO E&G) at the centre for radioisotopes in Kyushu University, Japan. The acquisition times were: 12,305 s for the KOI sample, using GMX30; 86,414 s for the OTZ sample, using GMX40; and 263,001 s for the AQC sample, using GMX23.

## Results

**Figure 1:**
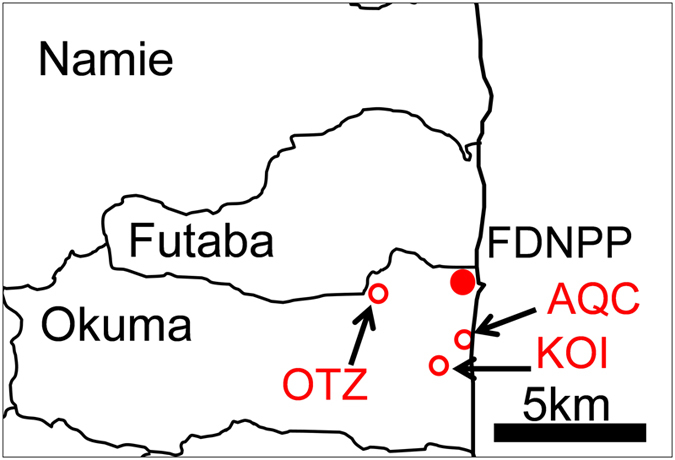
Locations of the samples used in this study. (This is the original product created by power point).

The CsMPs were discovered in three samples within ~4 km of the FDNPP: in gravel soil at the assembly house in Koirino, in soil from a side ditch at an aquaculture centre and in paddy soil in Ottozawa (Fig. 1). The samples are hereafter labelled KOI, AQC and OTZ, respectively. The radioactivity of the CsMPs and the relevant parameters are summarised in Table 1. The ^134^Cs/^137^Cs radioactivity ratio of the samples is 0.97–1.1, with an average of 1.04, which approximately corresponds to ~26 GWd/tU according to OrigenArp calculations^18^. Because of the heterogeneity within even the irradiated fuels in a single reactor, the source reactor unit could not be determined based only on the isotopic or radioactivity ratios. The radioactivity per unit mass of the CsMPs calculated assuming that the radioactivity for SiO_2_ glass^19^ with a density of 2.6 g/cm^3^ varies from 9.5 × 10^10^ to 4.4 × 10^11^ (Bq/g), which is comparable with values reported for CsMPs from Tokyo^20^.

**Figure 2:**
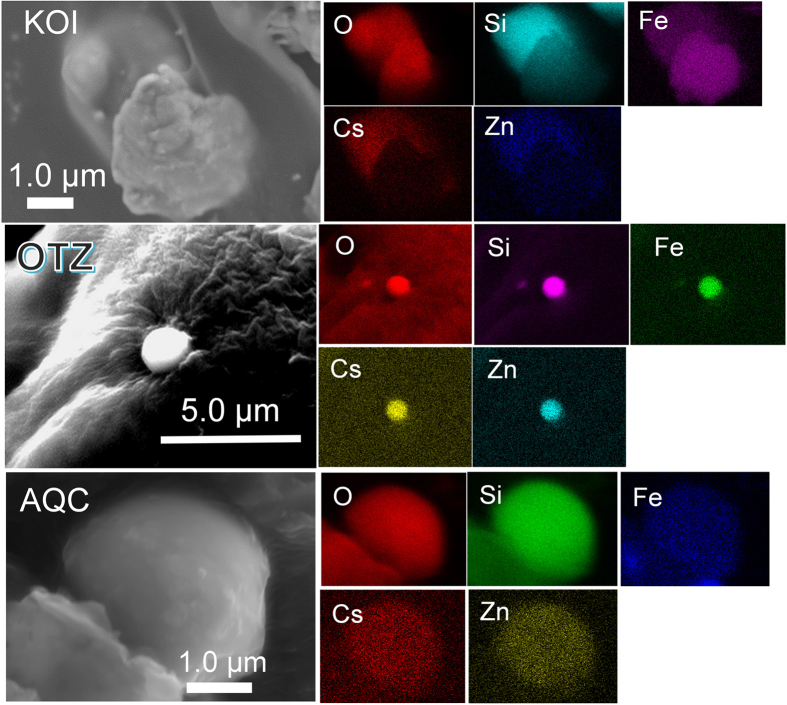
Secondary electron images of three CsMPs; KOI, OTZ, and AQC, associated with the energy dispersive X-ray spectrum (EDX) maps of the major constituents.

**Figure 3:**
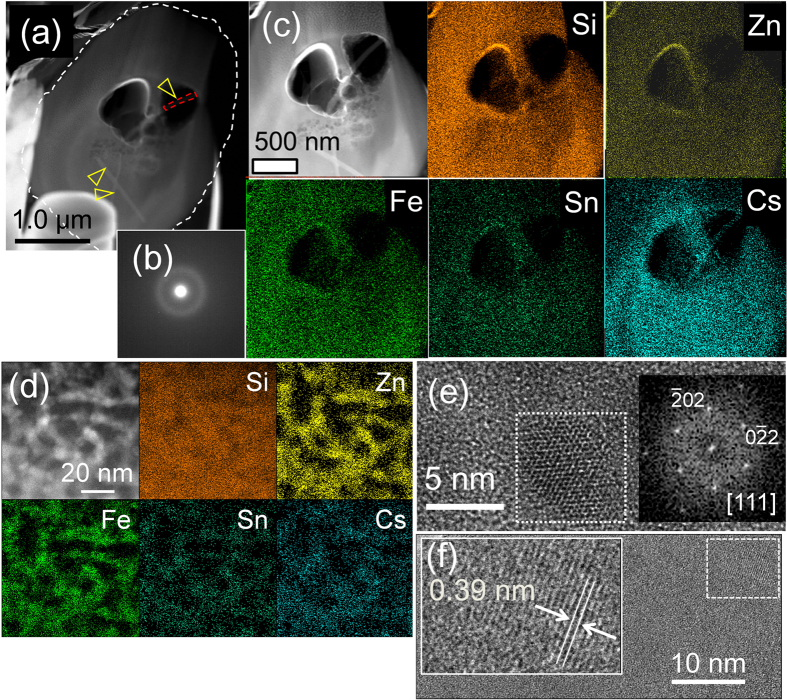
(**a**) HAADF-STEM image of the focussed ion beam (FIB)-prepared specimen of the KOI Cs-rich micro-particle, with its original shape traced by a white dotted line. The yellow and orange open triangles indicate rod-like nanoparticles consisting primarily of Cs. (**b**) SAED pattern of the area indicated in (**a**). (**c**) A HAADF-STEM image associated with elemental maps of the major constituents. (**d**) HAADF-STEM image with the elemental maps of the CsMP at high resolution, showing the heterogeneous occurrence of Fe–Zn-oxide nanoparticles associated with Sn and Cs. (**e**) HRTEM image of the Fe–Zn oxide and the fast Fourier transformed (FFT) image. (**f**) A HRTEM image of a rod-shaped Cs nanoparticle present in a pore indicated by the yellow arrow in (**a**).

The KOI CsMP was mainly composed of Si, Fe, Zn and Cs (Fig. 2 and Table S1). A HAADF-STEM image of the cross section shows two large pores of approximately 500 nm and numerous small pores in sizes ranging from 10–200 nm, indicating that some gases (such as H_2_, H_2_O, CO and CO_2_) were trapped through sparging during the MCCI (Fig. 3a). Selected area electron diffraction (SAED) patterns revealed diffuse diffraction maxima that correspond to an amorphous structure (Fig. 3b). Trace elements, including K, Cl, Sn, Rb, Pb and Mn, were detected by STEM energy dispersive X-ray (EDX) area analysis (Table S1).

**Figure 4:**
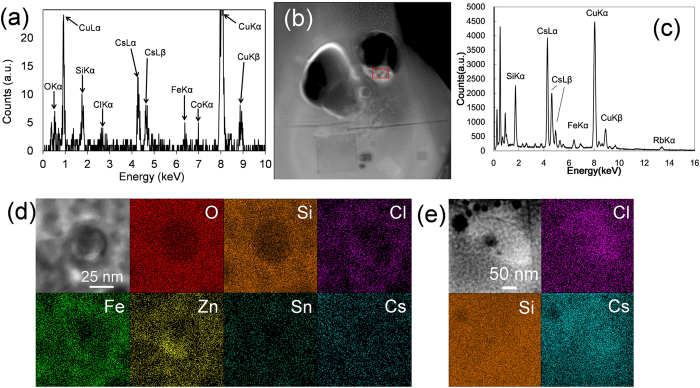
(**a**) The STEM-EDX spectrum of a rod-like nanoparticle shown in Fig. 3a. (**b**) HAADF-STEM image of the core region in the KOI obtained in the second session, showing that the rod-like Cs nanoparticle in the large pore degraded. (**c**) EDX spectrum of the area indicated by the red square in the left image. The composition of the degraded particle also revealed the Cs as the major constituent. (**d**) HAADF-STEM image of nano-sized inclusion of ZnCl_2_. (**e**) HAADF-STEM image of nano-sized inclusion of CsCl.

An elemental map of the CsMP constituents shows the synchronised distribution of Si, O, Fe and Zn, although only Cs is concentrated in the particle cores (Fig. 3c). Although the SAED exhibits diffuse diffraction maxima (Fig. 3b), a magnified image reveals that Zn, Fe, Sn and Cs are associated with nanoparticles as small as <10 nm distributed within the SiO_2_ matrix (Fig. 3d) that were identified to be franklinite structures (ZnFe_2_O_4_, *Fd*3m, Z = 8)^21^ (Fig. 3e). Several rod-like nanoparticles, indicated by yellow arrows, are present (Fig. 3a). An HRTEM image of the rod-shaped nanoparticle reveals a mostly amorphous contrast, with a small portion that is still crystalline (Fig. 3f). Based on the d-spacing in the HRTEM image (Fig. 3g) and the composition of primarily Cs and O (Fig. 4a–c), these rod-shaped particles were identified as Cs hydroxides, CsOH•H_2_O^22^. Nano-sized inclusions of ZnCl_2_ and CsCl were also identified (Fig. 4d,e).

**Figure 5:**
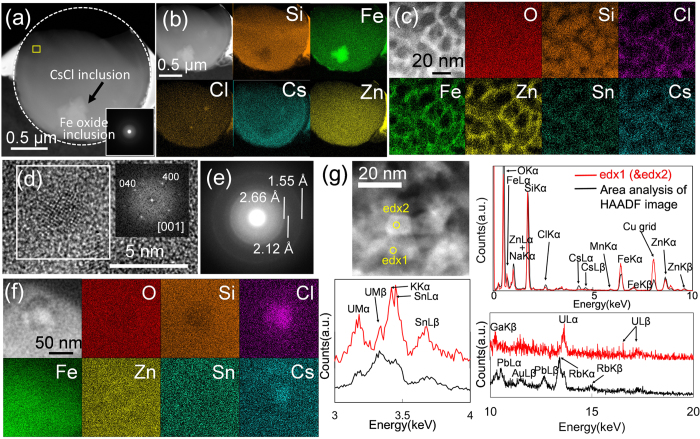
(**a**) HAADF-STEM image of the FIB-prepared OTZ CsMP. The inset is the SAED pattern obtained from the top thin area. White dotted curves represent the original shape of the particle before FIB thinning. (**b**) Elemental maps of the CsMP showing the distribution of major constituents. (**c**) Enlarged HAADF-STEM image with the elemental maps of major constituents, showing numerous Fe–Zn nanoparticles associated with Sn, Cs and Cl in the Si matrix. (**d**) Fe–Zn-oxide nanoparticle identified as franklinite. (**e**) SAED pattern exhibiting faint diffraction rings in diffuse halo, which are confirmed to be caused by franklinite. (**f**) HAADF-STEM image with elemental maps revealing the presence of CsCl domains. (**g**) HAADF-STEM image of the area indicated by the yellow square in (**a**) showing aggregation of franklinite nanoparticles. Comparison of edx spectra (edx1 and 2 in red line) of the point analysis with the spectrum obtained by the area analysis (black line) reveals the presence of U peaks.

In the OTZ CsMP, there are no pores, and the particle appears to have a homogeneous composition except for an Fe-oxide inclusion (Fig. 5a,b). However, like the KOI CsMP, the OTZ CsMP is composed of an amorphous SiO_2_ glass matrix along with Fe–Zn-oxide nanoparticles of <10 nm in size (Fig. 5c); these nanoparticles were identified as franklinite, based on the FFT image and SAED pattern (Fig. 5d,e). Franklinite was the only nanomaterial for which the structure was convincingly characterized. Caesium, Cl and Sn were associated with the franklinite for the most part; however, an inclusion of CsCl associated with ZnCl is also present (Fig. 5f). Remarkably, the area indicated by the yellow square in Fig. 5a contains nanoparticles with peaks of U *M*α, *L*α and *L*β in the EDX spectrum (Fig. 5g). Point analyses of the particles (edx1 and 2) exhibited further distinctive U peaks without interference from Rb (red line in the spectrum). The HAADF-STEM image resolved no UO_2_ crystal, only franklinite associated with a small amount of U.

**Figure 6:**
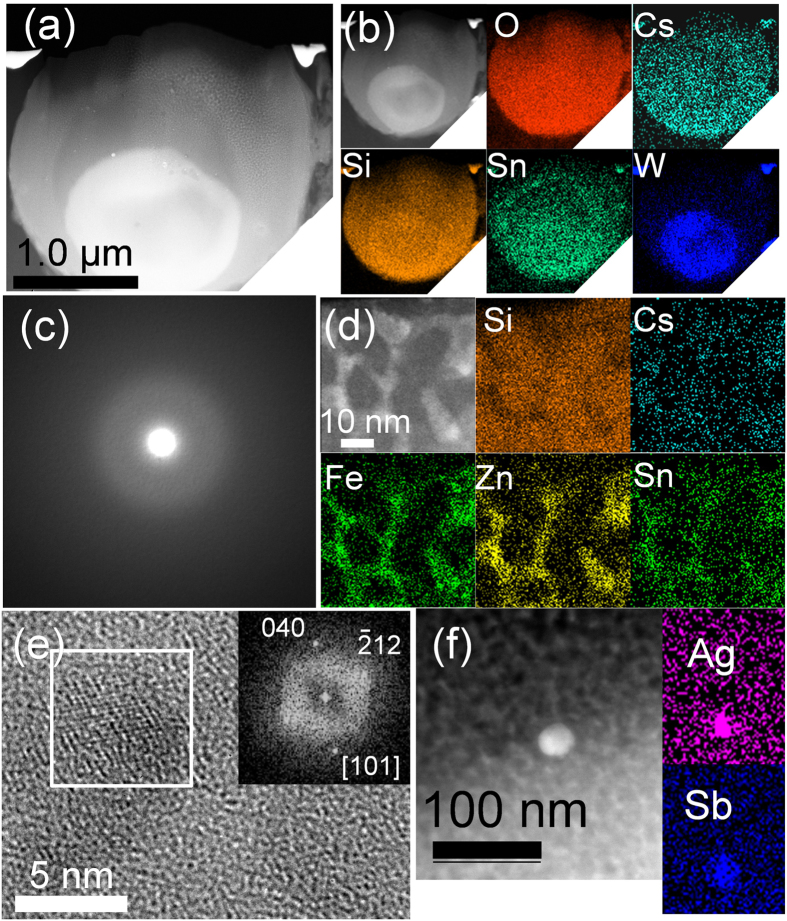
(**a**) A HAADF-STEM image of the cross-section of AQC CsMP prepared by FIB. (**b**) The STEM-EDX elemental maps of the major constituents. (**c**) SAED pattern of top thin area of FIB specimen shown in (**a**). (**d**) Magnified HAADF-STEM image of the thin area of FIB specimen associated with STEM-EDX elemental maps. (**e**) HRTEM image of a Zn–Fe oxide and the FFT image of the area outlined by the white square. (**f**) HAADF-STEM image of a fission product nanoparticle consisting of Ag and Sb.

The AQC CsMP exhibits a spherical shape (Fig. 6a) containing a spherical W oxide core as large as ~1 μm in diameter (Fig. 6b), which indicates that W oxide initially melted to form a droplet that served as a nucleation centre for CsMP formation. Otherwise, the AQC CsMP has a composition similar to that of the KOI and OTZ CsMPs, that is, Zn–Fe-oxide nanoparticles embedded in an SiO_2_ glass matrix (Fig. 6c–e). Some fission-product nanoparticles consisting of Ag and Sb were characterized in the CsMP as well (Fig. 6f).

**Figure 7:**
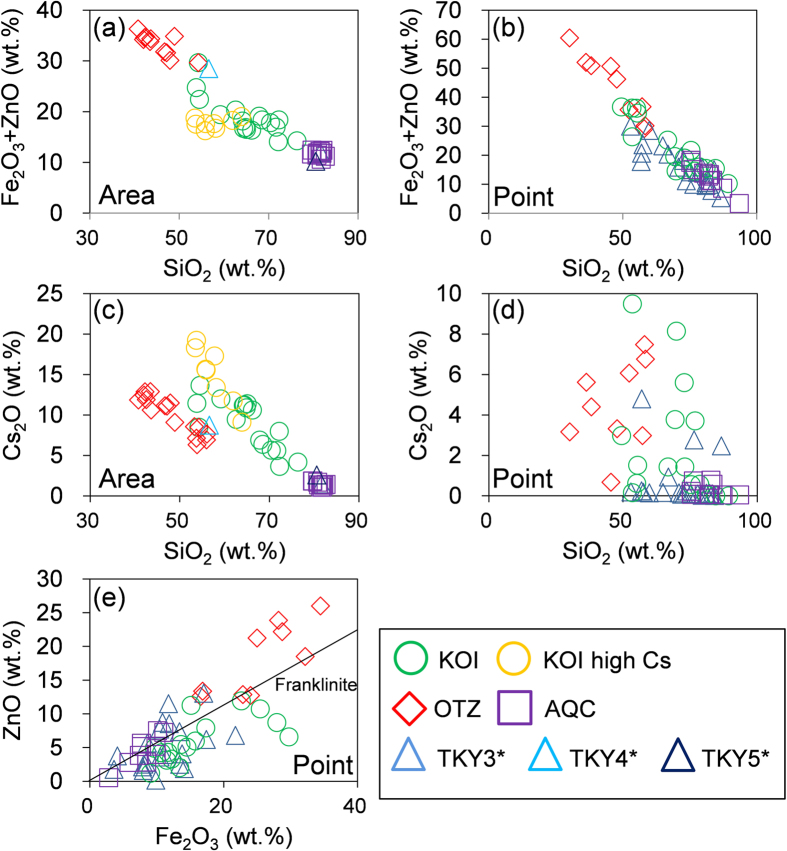
(**a**–**d**) Diagrams showing compositional relationship between Si and Fe + Zn (**a**,**b**), and between Si and Cs (**c**,**d**). (**a**,**c**): results of area analysis. (**b**,**d**): results of point analysis. (**e**) Correlation between Zn and Fe contents with a line of ideal composition of franklinite (ZnFe_2_O_4_). Correlations between all other elements measured in the CsMPs are given in Figs S2 and S3. *The TKY data are from Imoto *et al*. (2017)^20^.

The STEM-EDX area analysis (~100 × 100 μm) and point analyses of individual Zn–Fe oxides revealed that the Si concentrations are linearly correlated with the Zn + Fe content (Fig. 7a,b), indicating that the CsMPs are essentially composed of SiO_2_ glass and Fe–Zn-oxide nanoparticles, with the number of nanoparticles directly reflecting the concentrations of Fe and Zn. The Cs concentration derived from the area analysis also has a linear correlation with the Si content (Fig. 7c), whereas the Cs concentrations in the individual Fe–Zn oxide particles are scattered without correlation to the Si content (Fig. 7d). Such differences can be attributed to either variations in the concentration of Cs associated with Fe–Zn-oxide nanoparticles and/or intrinsic Cs species such as Cs(OH) and CsCl. Indeed, some area analyses of the KOI CsMP tended toward high Cs content (yellow circles) because of the presence of intrinsic Cs particles trapped inside the CsMP. The Zn concentration is positively correlated with Fe concentration towards the ideal Zn/Fe ratio of franklinite, as indicated by the solid line (Fig. 7e). The deviation toward a higher amount of Zn is a result of the presence of ZnCl_2_ inclusions.

## Discussion

As was shown in previous experiments^15^,^23^,^24^, the occurrence of Cs and other FP nanoparticles strongly suggests that volatile FPs (Cs, I, Xe, Te, Ag and Rb)^9^, which accumulated in the gap between the fuel and the cladding during reactor operation, were released either immediately after cladding failure or during the melting of the fuel rods in the FDNPP prior to MCCI. Thus, the atmosphere inside the RPVs must have been filled with aerosol particles associated with Cs, gaseous Cs species, water vapour and hydrogen gas. Interactions between the melted core and Fe in the structural materials of the reactor during vessel failure then produced large amounts of Fe–Zn-oxide nanoparticles.

As recent results^20^,^25^ showed, the major and trace elements of the CsMPs were derived from elements inside the reactor during the meltdowns; however, the compositions are markedly different from those in the debris^12^, which consists of a mixture of melted core, reactor materials and concrete. Possible sources of the constituent elements are as the follows: Sn was part of a Zr–Sn alloy; Fe and Mn were constituents of the reactor pressure vessel; Si was derived from siliceous concrete released during the molten-core–concrete interaction^3^,^23^; Cs, Rb, Pb and Sn were fission products contained in the irradiated fuels; Cl was from seawater and Zn was routinely added to the reactor water to prevent radioactive corrosion of steel by formation of a protective oxide layer. Tungsten is present as an impurity in zircaloy and most stainless steels^26^. Although W is a promising element with extremely high heat resistance (a melting temperature of 3687 K), the oxidized form can be easily melted at relatively low temperature of ~1746 K^19^. It is plausible that the presence of water vapour dramatically enhanced the oxidation of the stainless steel^26^.

As reported in the MCCI experimental study^22^,^23^, when the melted cores hit the siliceous concrete pedestal, SiO(g) was generated as a consequence of Zr oxidation at a temperature >2,143 K. In this study, it was found that SiO(g) eventually condensed to SiO_2_ glass rather than forming Si metal or SiC, indicating that there was some oxygen within the reactor PCVs at the FDNPP. The presence of oxygen affects the volatilization temperature of radionuclides in the fuel such as U^27^. The oxidised form of U oxide fuel can volatilise at ~1,900 K by 10% of the total UO_2_, whereas for non-oxidized form of UO_2_ the figure is nearly 0% at 2,700 K^28^. Thus, it is plausible that the trace amounts of U associated with franklinite are evidence of the volatilisation of slightly oxidised UO_2_. The absence of UO_2_ fragments in the CsMPs suggest that fuel fragments were not directly incorporated into the CsMPs during the MCCI.

The airborne CsOH nanoparticles that formed in the PCVs prior to MCCI were trapped during the MCCI events. Considering that CsOH is stable as a solid at temperatures <615 K^19^, which is much lower than the temperature of SiO_2_ solidification, ~1995 K^19^, it is likely that the CsMP rapidly cooled and solidified without degrading the CsOH particles, which is consistent with the glassy structure of the SiO_2_ matrix. The CsOH particles might have decomposed if they were trapped in pores that contained water vapour and then recrystallized while the CsMP cooled down.

In addition, at the time of MCCI, the other gases (H_2_, H_2_O, CO and CO_2_) are typically sparged from the molten corium pool and must have been trapped in the micro-particles during the condensation of SiO(g). The trapped gases created the porous texture, and the SiO_2_(l) rapidly solidified into glassy SiO_2_, thus retaining numerous pores. Thus, the pore found within the KOI CsMPs was probably filled with CO_2_ and water vapour, in addition to possible gaseous decay daughters, due to the oxidizing conditions. The difference in the micro-texture with (KOI) or without (OTZ and AQC) pores possibly represents a local variation in the amount of vapours trapped during condensation of the SiO(g).

The formation process of CsMPs in the FDNPP was clearly different from that of the micro-particles reported in the previous MCCI experiments^23^,^29^,^30^. The nanoscale textures of the CsMPs revealed several processes during meltdown: (*i*) FPs, such as Cs, were released to form nanoparticles or were present in mist droplets during the meltdown; (*ii*) many Zn–Fe-oxide nanoparticles formed during the failure of the RPVs, and Cs dissolved in mist droplets attached to the surfaces of airborne Zn–Fe-oxide nanoparticles; (*iii*) the molten fuels that melted through the RPVs hit the concrete pedestal and generated SiO gas at >2,000 K, which immediately condensed as SiO_2_ over the Zn–Fe-oxide nanoparticles and incorporated the FP nanoparticles.

A recent study reported interesting phenomena during a laboratory experiment involving CsOH adsorption onto a stainless-steel surface at an elevated temperature^31^. The authors found that CsOH can easily adsorb onto an Fe-oxide surface. Their results are consistent with our results revealing a close association of CsOH with Fe–Zn-oxide nanoparticles. However, the resulting product of chemisorption, CsFeSiO_4_, which was characterized in their study, was not observed in the present study; Si occurs as pure SiO_2_. The difference strongly suggests that the CsMP did not form in the process where the melted fuel encountered the material of the RPV, but instead formed via another reaction process, most likely interaction with the concrete pedestal, as suggested in the present study.

As a recent study reported that ~90% of the Cs radioactivity derived from CsMPs during the initial fallout of radioactive Cs in Tokyo^20^, CsMPs with FP nanoparticles are significant sources of radioactive Cs and FPs for the surface environment in Fukushima. Although the contribution of the CsMPs to the total inventory of radioactivity in the contaminated area in Fukushima remains to be determined, the nanoscale physical and chemical properties of the CsMPs provide clues for understanding the mechanisms of Cs release and the stability of Cs after dispersal to the environment. Although their total activity is low, CsMPs are yet another vector for the dispersion of low-volatility radionuclides, such as U, in addition to volatile radionuclides, to the surrounding environment. Thus, the migration of CsMPs in the environment should be taken into account in the Cs transport model of the Fukushima environment in order to gain a better understanding of the impact and dynamics of radionuclide contamination.

## Conclusions

The sequence of chemical and physical processes inside the reactors during the meltdowns in the FDNPP have been unravelled based on state-of-the-art atomic-resolution electron microscopy of CsMPs. The CsMPs are as small as a few microns and comprise SiO_2_ glass matrices and ~10 nm-sized Zn–Fe-oxide nanoparticles associated with up to ~20 wt% of Cs, occasionally accompanied by trace amounts of U. The micro-texture of the CsMPs reveals that various airborne fission-product nanoparticles were first released from the fuels before and during meltdowns. Subsequently, RPV failure occurred and a large number of Zn–Fe-oxide nanoparticles were produced. Finally, the melted core interacted with concrete and the MCCI proceeded via SiO_2_ condensation encompassing the Zn–Fe-oxide nanoparticles, incorporating the fission-product nanoparticles. The present study demonstrates that the CsMPs provide an important clue for understanding the reactions and conditions inside the reactors. On the other hand, because of the extremely high radioactivity per unit mass, ~10^11^ Bq/g, CsMPs can be a significant source of the radiation dose in the ambient environment in Fukushima. In addition, CsMPs are an important carrier by which volatile and low-volatility radionuclides such as U reach the environment.

## Additional Information

**How to cite this article**: Furuki, G. *et al*. Caesium-rich micro-particles: A window into the meltdown events at the Fukushima Daiichi Nuclear Power Plant. *Sci. Rep.* **7**, 42731; doi: 10.1038/srep42731 (2017).

**Publisher's note:** Springer Nature remains neutral with regard to jurisdictional claims in published maps and institutional affiliations.

## Supplementary Material

Supplementary Information

## Figures and Tables

**Table 1:** Summary of the radioactivity and the associated parameters of three CsMPs in the present study.

Sample	Particle size (μm)	Radioactivity (Bq)*		^134^Cs/^137^Cs	Radioactivity per unit mass (Bq/g)**	Cs concentration by TEM-EDX (wt.%)
		^134^Cs	^137^Cs			
KOI	3.4	12.4(±0.36)	11.3(±0.15)	1.10	4.43 × 10^11^**	3.67–19.2
OTZ	2.0	2.00(±0.080)	2.07(±0.031)	0.967	3.74 × 10^11^**	8.50–12.9
AQC	3.3	0.940(±0.013)	0.906(±0.040)	1.04	3.95 × 10^10^**	1.09–1.87

The radioactivities of ^134^Cs and ^137^Cs were given, as the gamma spectroscopy of these individual particles revealed only peaks of ^134^Cs and ^137^Cs. *The radioactivity was decay-corrected to March 12, 2011, 15:36 JST. **The radioactivity per unit mass was calculated assuming that the particles have spherical shape and the density is 2.6 g/cm^3^.
