# Supplementary material for: Caesium-rich micro-particles: A window into the meltdown events at the Fukushima Daiichi Nuclear Power Plant
Source: Sci Rep. 2017 Feb 15;7:42731. doi: 10.1038/srep42731 (PMC5309886; doi:10.1038/srep42731)
Supplement: Supplementary Information [file srep42731-s1.pdf]

Supplementary Material for

Cesium-rich micro-particles: A window into the meltdown events at the Fukushima Daiichi Nuclear Power Plant

By, Genki Furuki, Junpei Imoto, Asumi Ochiai, Shinya Yamasaki, Kenji Nanba, Toshihiko Ohnuki, Bernd Grambow, Rodney C. Ewing, and Satoshi Utsunomiya*

In *Scientific Reports*

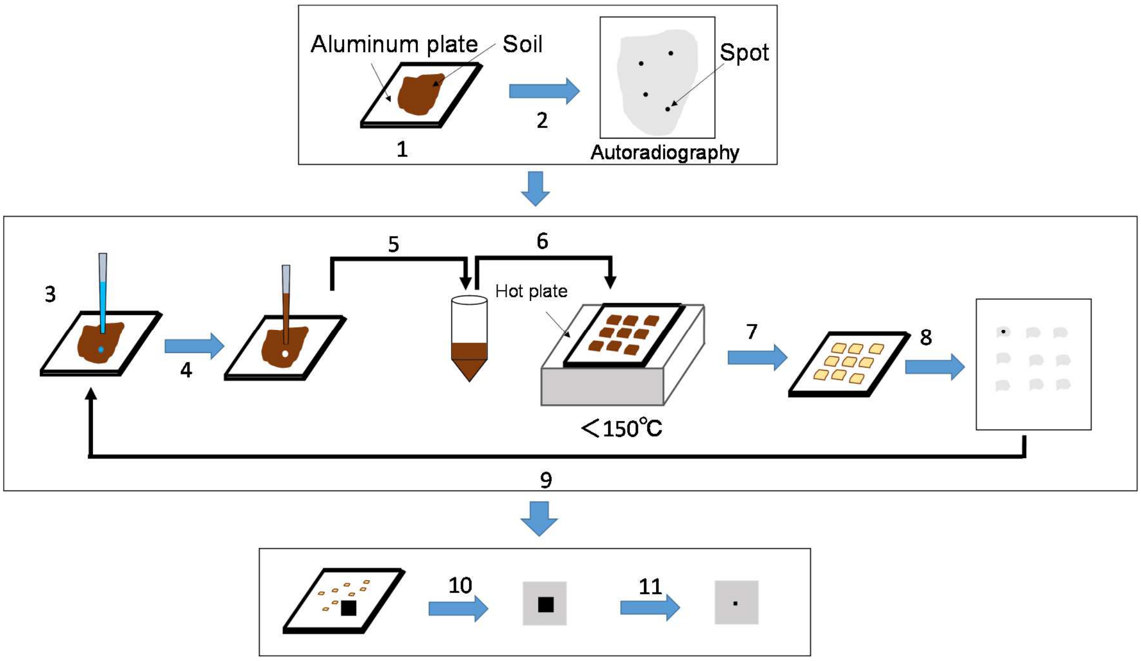

Figure S1. A diagram showing the procedure separating CsMPs from the soil samples.

Table S1. Chemical composition (wt.%) of the area analysis on the FIB thin section of the KOI, OTZ, and AQC CsMPs as determined by STEM-EDX standardless quantification. The analysis was conducted using a rastered STEM probe (~100×100 nm) in order to minimize the diffusion of alkaline elements, such as K and Cs, under the electron beam. The composition was calculated as an oxide except for Cl and normalized to 100% in total. Iron concentration is presented as Fe_2O_3.

	SiO_2	Fe_2O_3	ZnO	Cs_2O	SnO_2	Rb_2O	K_2O	Cl	MnO	PbO
KOI										
	71.6	11.3	5.63	5.63	4.11	0.40	0.11	0.69	0.34	0.15
	72.2	9.39	4.66	8.00	3.69	0.87	0.37	0.61	0.08	0.17
	76.3	9.34	4.92	4.20	3.49	0.60	0.18	0.48	0.19	0.27
	66.2	10.1	6.30	10.6	3.46	1.55	0.44	0.70	0.24	0.44
	70.4	11.3	6.52	5.66	4.31	0.00	0.46	0.78	0.54	0.00
	67.8	13.3	5.83	6.92	4.61	0.00	0.24	0.70	0.59	0.00
	72.3	11.4	6.97	3.67	3.92	0.00	0.22	0.74	0.82	0.00
	54.3	17.3	12.3	8.50	1.95	2.08	0.95	1.72	0.87	0.01
	68.5	11.5	6.87	6.38	4.29	0.62	0.50	0.73	0.47	0.11
	64.4	10.4	6.22	11.3	4.28	1.32	0.92	0.85	0.31	0.00
	65.0	10.8	5.67	11.4	3.82	1.71	0.37	0.84	0.33	0.01
	64.8	10.7	6.19	10.9	4.35	1.47	0.29	0.59	0.39	0.36
	59.20	12.3	7.12	12.0	4.47	2.47	0.49	0.83	0.46	0.72
	64.1	12.0	6.17	11.2	3.24	1.70	0.39	0.77	0.40	0.01
	53.8	18.6	6.12	11.5	6.38	1.31	0.11	1.02	0.85	0.36
	62.6	12.8	7.53	9.47	4.22	1.23	0.46	0.66	0.59	0.47
	54.5	15.6	6.81	13.7	4.73	2.01	0.48	0.81	0.71	0.68
high KOI										
	61.9	11.8	6.47	11.7	3.74	1.73	0.69	0.80	0.47	0.67
	57.8	11.1	6.55	17.3	3.71	1.42	0.40	0.61	0.43	0.72
	53.7	11.1	6.38	19.2	3.58	3.31	1.20	0.63	0.47	0.35
	53.5	11.9	6.83	18.3	4.46	2.43	1.03	0.61	0.53	0.41

	55.9	11.9	5.70	15.6	4.02	2.96	1.29	2.08	0.56	0.00
	58.1	11.8	4.88	13.4	3.80	3.19	1.09	3.14	0.40	0.14
	55.8	11.8	4.45	15.7	3.70	2.96	1.27	3.25	0.35	0.80
	63.9	12.2	6.84	9.12	3.68	1.12	0.76	0.85	0.57	0.97
OTZ										
	48.9	20.5	14.3	9.09	2.29	0.00	1.80	2.24	0.85	0.00
	54.3	17.3	12.3	8.50	1.95	2.08	0.95	1.72	0.87	0.01
	46.7	18.1	13.7	11.2	2.8	2.30	2.04	1.91	0.88	0.46
	47.9	16.9	13.2	11.5	2.32	2.34	2.23	1.52	1.01	1.12
	43.6	19.1	15.3	10.6	2.27	2.26	2.45	1.95	1.05	1.44
	47.1	17.8	13.8	11.1	2.85	2.07	1.69	1.6	0.84	1.11
	43.4	19.5	14.3	12.9	2.95	2.36	1.70	1.48	0.84	0.53
	42.5	19.7	15.1	11.9	2.16	2.53	2.41	1.75	0.99	0.98
	42.2	19.6	15.0	12.9	2.57	2.74	1.93	1.61	1.06	0.43
	42.0	19.3	15.0	12.5	3.15	2.80	1.75	1.58	1.04	1.01
	40.8	20.7	15.6	11.9	2.43	2.75	2.45	1.73	1.12	0.61
AQC										
	80.8	6.08	5.34	1.71	3.31	0.00	2.38	0.33	0.08	0.00
	82.7	6.36	4.82	1.42	3.30	0.00	1.04	0.41	0.00	0.00
	81.5	7.08	5.08	1.12	3.66	0.00	1.24	0.37	0.00	0.00
	81.0	6.36	5.48	1.37	3.23	0.00	2.01	0.38	0.21	0.00
	79.7	6.61	5.80	1.87	3.33	0.00	2.06	0.37	0.26	0.00
	81.6	6.37	5.76	1.36	3.09	0.00	1.11	0.41	0.35	0.00
	82.1	6.28	6.08	1.34	2.58	0.00	0.90	0.43	0.27	0.00

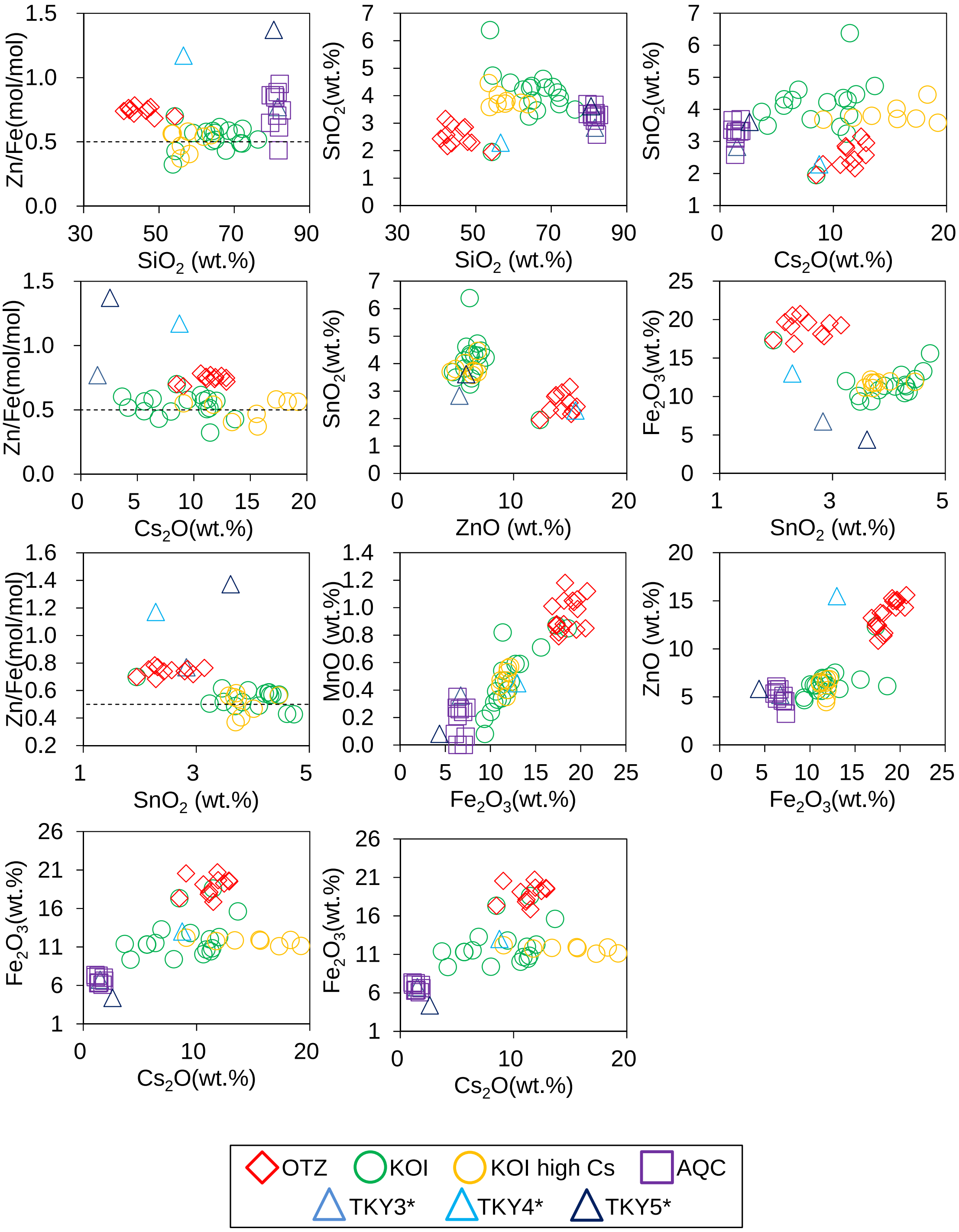

Figure S2. Diagrams showing the correlation between the constituent elements of the CsMPs based on the STEM-EDX area analysis (wt.%). *The TKY data are from Imoto *et al.* (2017)[20].

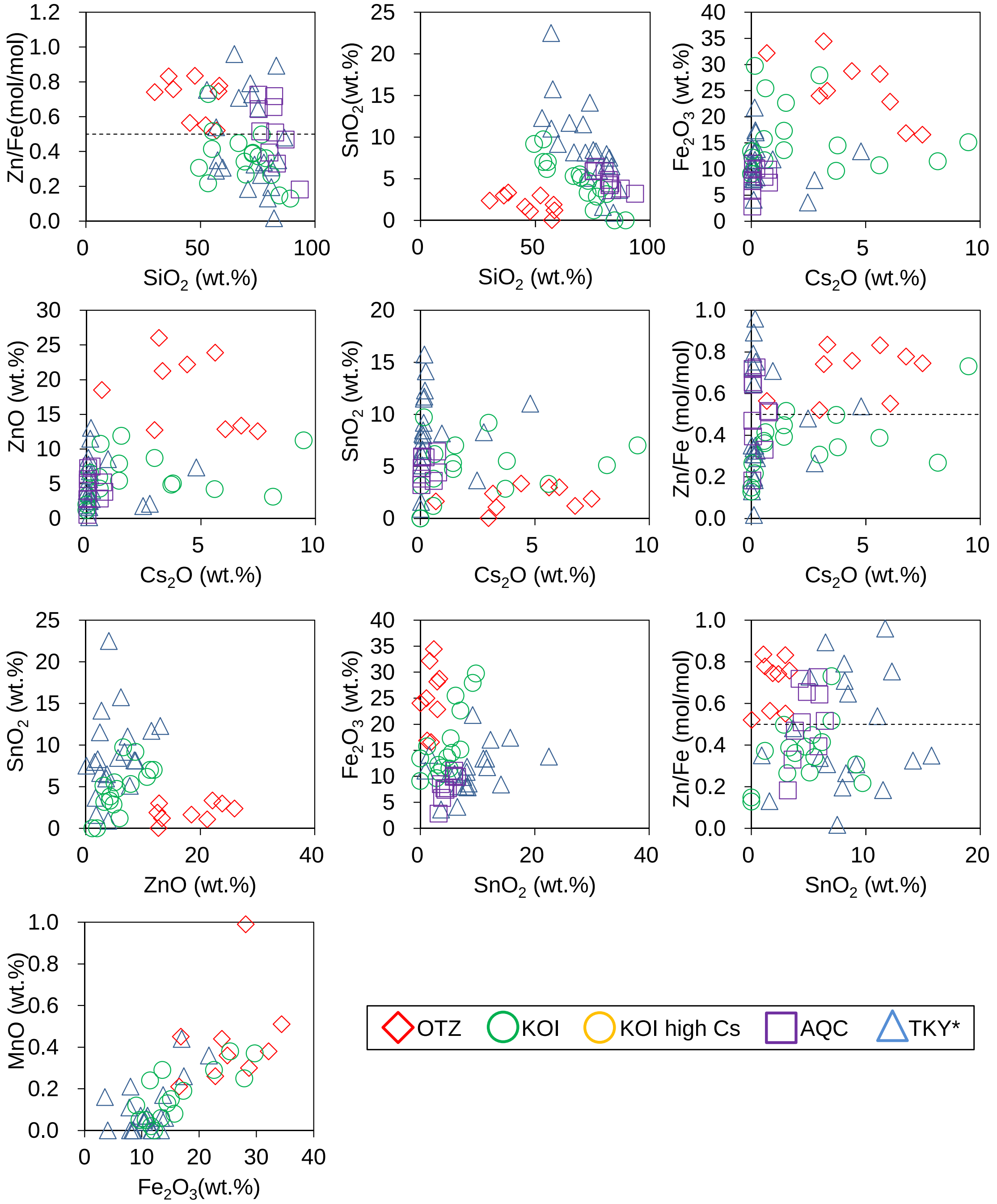

Figure S3. Diagrams showing the correlation between the constituent elements of the CsMPs based on the STEM-EDX point analysis (wt.%). * The TKY data from Imoto *et al.* (2017)[20].
